# ﻿Taxonomy and evolution history of two new litter-decomposing *Ciliochorella* (Amphisphaeriales, Sporocadaceae)

**DOI:** 10.3897/mycokeys.100.108863

**Published:** 2023-11-13

**Authors:** Jia-Yu Song, Hai-Xia Wu, Jin-Chen Li, Wei-Feng Ding, Cui-Ling Gong, Xiang-Yu Zeng, Nalin N. Wijayawardene, Da-Xin Yang

**Affiliations:** 1 International Fungal Research and Development Centre, Institute of Highland Forest Science, Chinese Academy of Forestry, Kunming 650224, China International Fungal Research and Development Centre, Institute of Highland Forest Science, Chinese Academy of Forestry Kunming China; 2 Key Laboratory of Breeding and Utilization of Resource Insects, National Forestry and Grassland Administration, Kunming 650224, China Key Laboratory of Breeding and Utilization of Resource Insects, National Forestry and Grassland Administration Kunming China; 3 Department of Plant Pathology, College of Agriculture, Guizhou University, Guiyang 550025, China Guizhou University Guiyang China; 4 Centre for Yunnan Plateau Biological Resources Protection and Utilization, College of Biological Resource and Food Engineering, Qujing Normal University, Qujing, Yunnan 655011, China Qujing Normal University Qujing China; 5 Kunming Branch (KMB), Chinese Academy of Sciences (CAS), Kunming, Yunnan 650204, China Kunming Branch (KMB), Chinese Academy of Sciences (CAS) Kunming China

**Keywords:** Ancestral biogeography, leaf litter degradation, morphology, new taxa, time dating

## Abstract

The genus *Ciliochorella* is a group of pestalotioid fungi, which typically occurs in subtropical and tropical areas. Species from the *Ciliochorella* genus play important roles in the decomposition of litter. In this study, we introduce two new species (*Ciliochorellachinensis***sp. nov.** and *C.savannica***sp. nov.**) that were found on leaf litter collected from savanna-like vegetation in hot dry valleys of southwestern China. Phylogenetic analyses of combined LSU, ITS and *tub2* sequence datasets indicated that *C.chinensis* and *C.savannica* respectively form a distinct clade within the *Ciliochorella* genus. The comparison of the morphological characteristics indicated that the two new species are well differentiated within this genus species. Analysis of the evolutionary history suggests that *Ciliochorella* originated from the Eurasian continent during the Paleogene (38 Mya). Further, we find that both new species can produce cellulase and laccase, playing a decomposer role.

## ﻿Introduction

Fungi thrive on diverse ecosystems and environments as pathogens, mutualists, and saprobes (Bucher et al. 2004; Schmit and Mueller 2007; Jobard et al. 2010; Hyde et al. 2020; Lai et al. 2021). As decomposers of nature, fungi are involved in the degradation of lignin and cellulose (Bisaria and Ghose 1981; Hatakka 1994; Adlini et al. 2014). The extracellular lignin-degrading enzymes of fungi mainly comprise two types, peroxidases and laccases (Pointing et al. 2005; Floudas et al. 2012). Some plant-specific pathogenic fungi use laccases to counter the effects of tannic acid, which also has anti-viral activities (Dong and Changsun 2021). As such, laccase activity is also considered a virulence factor in many fungal diseases.

*Ciliochorella* Sydow & Mitter (1935), typified by *C.mangiferae* Syd., is an important genus of pestalotioid fungi (Sutton 1980; Nag Raj 1993; Lee et al. 2006; Tanaka et al. 2011; Tangthirasunun et al. 2015; Wijayawardene et al. 2016; Liu et al. 2019a). Most taxa classified as pestalotioid fungi are phytopathogens that cause a variety of diseases in plants, some of which are saprobes or endophytes that are widely distributed in tropical and temperate regions (Benjamin and Guba 1961; Barr 1975; Nag Raj 1993; Maharachchikumbura et al. 2016). Liu et al. (2019a) and Wijayawardene et al. (2022a) placed this genus in Sporoca­daceae (Ascomycota, Sordariomycetes, Amphisphaeriales).

*Ciliochorella* is an asexually typified, coelomycetous genus with nine species listed in the Index Fungorum (2023). However, only six species are accepted in the Species Fungorum (2023) due to the following two reports: 1) Subramanian and Ramakrishnan (1956) proposed *Shanoria* Subram. & Ramakr., a new genus, to accommodate *Ciliochorellabambusarum* Shanor as *Shanoriabambusarum* (Shanor) Subram. & K. Ramakr; 2) Nag Raj (1993) synonymized *Ciliochorellaeucalypti* T.S. Viswan. and *Ciliochorellaindica* Kalani under *C.mangiferae*). The genus is characterized by cylindrical, straight, or slightly curved conidia with septate pale brown middle cells, and colorless end cells bearing a single, eccentric appendage (Nag Raj 1993; Allegrucci et al. 2011; Hyde et al. 2016; Wijayawardene et al. 2016).

There are few studies that focused on the divergence time estimation of *Ciliochorella*, whereas some published studies are based on a larger classification scale (such as the order and the class). Divergence time can provide insights into the history of a given group of fungi species and its taxonomic placement (Li et al. 2005; Vijaykrishna et al. 2006; Beimforde et al. 2014; Pérez-Ortega et al. 2016; Samarakoon et al. 2016; Li et al. 2023). Samarakoon et al. (2016; 2022) estimated the divergence time of the Xylariomycetidae, including one species of *Ciliochorella* (*C.mangiferae*), diverged approximately 201–252 Mya in the Early Mesozoic (Samarakoon et al. 2022). Chen et al. (2023) conducted detailed genomic studies on the divergence time of members in Sordariomycetes, which included nine species of Sporocadaceae. The results show that the divergence time of Sporocadaceae is about 90.56 Mya. In addition to estimating divergence times, ancestral state reconstruction is also a viable strategy for studying evolutionary history. Ancestral state reconstruction can reveal the origin and evolution of a species and provide a basis for species classification (Omland 1999; Ismail et al. 2016; Royer-Carenzi and Didier 2016; Gao and Wu 2022; Li et al. 2022). However, relevant research examining the ancestral reconstruction of *Ciliochorella* is lacking.

Studies on the litter decomposition of *Ciliochorella* have demonstrated oxidative enzymatic activity using *in-vitro* cultures of *Ciliochorellabuxifolia* demonstrated by Troncozo et al. (2015). It is also worth noting that *C.mangiferae* is an important litter-decomposing taxon in tropical countries, especially in India (Masilamani and Muthumary 1994). This genus may be involved in the leaf decomposition process, but not all species in this genus have been verified to have this function (Saparrat et al. 2010).

The primary objectives of this study were: 1) to delineate the taxonomic status of newly collected *Ciliochorella*-like species; 2) to estimate the evolutionary history of *Ciliochorella*; and 3) to determine the litter-decomposing function of this genus species in nature based on the screening of cellulase and laccase production.

## ﻿Methods

### ﻿Morphological studies

Two *Ciliochorella*-like taxa were collected from leaf litter (dead leaves from an unidentified plant species) in the savanna-like vegetation of hot dry valleys in southwestern China. The samples were placed in paper bags and transported to the laboratory for further observation. Following Wu et al. (2014), the collected samples were processed and examined by microscopes: photographs of ascomata were taken by using a compound stereomicroscope (KEYENCE CORPORATION V.1.10 with camera VHZ20R). Hand sections were made under a stereomicroscope (OLYMPUS SZ61) and mounted in water and blue cotton Photomicrographs of fungal structures were taken with a compound microscope (Nikon ECLIPSE 80i).

The images used for the figures were processed using the software Adobe Photoshop CC v. 2015.5.0 software (Adobe Systems, San Jose, CA, USA).

The specimens were deposited in the herbarium of IFRD (International Fungal Research & Development Centre; Institute of Highland Forest Science, Chinese Academy of Forestry, Kunming, China) and the cultures were deposited in the International Fungal Research & Development Center Culture Collection (IFRDCC) at the Research Institute of Highland Forest Science, Chinese Academy of Forestry, Kunming, China.

Single spore isolation was performed following the procedure published by Choi et al. (1999) and Chomnunti et al. (2014). Germinated spores were individually transferred to potato dextrose agar (PDA) medium and incubated at 26 °C for 48 h. Colony characteristics were observed and measured after two weeks at 26 °C.

Newly introduced taxa were registered at Fungal Names (https://nmdc.cn/fungalnames/) and obtained identifiers.

### ﻿DNA isolation, amplification and sequencing

Genomic DNA was extracted from mycelia growing on PDA at room temperature using the Forensic DNA Kit (OMEGA, USA) according to the manufacturer’s instructions. The primers LR0R and LR5 were used to amplify the 28S large subunit (LSU) rDNA (Vilgalys and Hester 1990). The internal transcribed spacer (ITS) rDNA was amplified and sequenced with the primers ITS5 and ITS4 (White et al. 1990). The primers T12 and T22 were used to amplify the β-tubulin (*tub2*) (O’Donnell and Cigelnik 1997). The following PCR protocol was used: initial denaturation at 98 °C for 2 min, then 30 cycles, i) 98 °C denaturation for 10 s, ii) 56 °C annealing for 10 s, and iii) 72 °C extension for 10 s (ITS) or 20 s (LSU and *tub2*) followed by a final extension at 72 °C for 1 min. All PCR products were sequenced by Biomed (Beijing, China).

### ﻿Sequence alignments and phylogenetic analyses

BioEdit version 7.0.5.3 was used to re-assemble sequences generated from forward and reverse primers to obtain the integrated sequences (Hall 1999). The sequences used by literature and closely related taxa from NCBI BLAST results (Table 1). Sequence alignments were performed in MAFFT (https://mafft.cbrC.jp/alignment/server/) (Katoh et al. 2019), and alignments were manually adjusted where necessary using BioEdit version 7.0.5.3. The sequence data set for subsequent analyses were obtained with the sequence fragments using R-based ape package (Paradis and Schliep 2019).

**Table 1.** Selected taxa in this study with their corresponding GenBank accession numbers and distribution information.

Species	Location	Voucher/ Strains	GenBank accession numbers			Reference
			LSU	ITS	*tub2*	
*Ciliochorellacastaneae*	East Asia (Japan); South Asia (India)	HHUF 28799	AB433277	–	–	Nag Raj 1993; Endo et al.2008
*C.castaneae*	East Asia (Japan); South Asia (India)	HHUF 28800	AB433278	–	–	Nag Raj 1993; Endo et al. 2008
***C.chinensis***	**East Asia (China)**	**IFRD 9468**	**OP902256**	**OP902250**	**OQ918680**	**In this study**
*C.dipterocarpi*	Southeast Asia (Thailand)	MFLUCC 22-0132	OP912990	OP912991	–	Nethmini et al. 2023
C.mangiferae*	Southeast Asia (Thailand); South Asia (India, Pakistan); America (Cuba); Africa (Nigeria, Sierra Leone);	MFLUCC 12-0310	KF827445	KF827444	KF827478	Nag Raj 1993; Masilamani and Muthumary 1994; Tangthirasunun et al. 2015
*C.phanericola*	Southeast Asia (Thailand)	MFLUCC 14-0984	KX789681	KX789680	KX789682.1	Hyde et al. 2016
***C.savannica***	**East Asia (China)**	**IFRD 9467**	**OP902279**	**OP902251**	**OQ926205**	**In this study**
	**East Asia (China)**	**IFRD 9473**	**OQ867459**	**OQ867475**	**OQ926206**	**In this study**
Discosiaaff.brasiliensis	Unknown	NBRC 104199	AB593707	AB594775	AB594185	Tanaka et al. 2011
D.aff.pleurochaeta	Unknown	KT2188	AB593713	AB594781	AB594179	Tanaka et al. 2011
D.artocreas*	Unknown	NBRC 8975	AB593705	AB594773	AB594172	Tanaka et al. 2011
*D.brasiliensis*	Southeast Asia (Thailand)	NTCL095	KF827437	KF827433	KF827470	Tangthirasunun et al. 2015
*D.celtidis*	East Asia (China)	MFLU 18-2581	MW114406	NR_174839	–	Tennakoon et al. 2021
*D.fagi*	Europe (Italy)	MFLU 14-0299A	KM678048	KM678040	–	Li et al. 2015
*D.fici*	East Asia (China)	MFLU 19-2704	MW114409.1	NR_174840	–	Tennakoon et al. 2021
*D.italica*	Europe (Italy)	MFLU 14-0298C	KM678044	KM678041	–	Li et al. 2015
*D.macrozamiae*	Oceania (Australia)	CPC 32113	MH327855	MH327819	MH327894	Crous et al. 2018
*D.pini*	Unknown	MAFF 410149	AB593708	AB594776	AB594174	Tanaka et al. 2011
*D.pseudoartocreas*	Europe (Austria)	CBS 136438	MH877640	NR_132068	MH554672	Crous et al. 2013
	Unknown	DUCC5154	MH844788	MH844763	–	Tanaka et al. 2011
*D.querci*	East Asia (China)	MFLU 18-0097	MW114405	MW114326	–	Tennakoon et al. 2021
*D.tricellularis*	Unknown	NBRC 32705	AB593728	AB594796	AB594188	Tanaka et al. 2011
*D.yakushimensis*	East Asia (Japan)	MAFF 242774	AB593721	AB594789	AB594187	Tanaka et al. 2011
*Discostromatosta*	Unknown	HKUCC 1004	AF382380	–	–	Tang et al. 2007
*Discost.fuscellum*	Europe (Italy)	MFLUCC 14-0052	KT005514	KT005515	–	Senanayake et al. 2015
*Discost.stoneae*	Unknown	NBRC 32690	AB593729	AB594797	–	Tanaka et al. 2011
Immersidiscosiaeucalypti*	East Asia (Japan)	NBRC 104195	AB593722	AB594790	–	Tanaka et al. 2011
I.eucalypti*	East Asia (Japan)	NBRC 104196	AB593723	AB594791	–	Tanaka et al. 2011
	East Asia (Japan)	NBRC 104197	AB593724.1	AB594792	–	Tanaka et al. 2011
	East Asia (Japan)	MAFF 242781	AB593725	AB594793	–	Tanaka et al. 2011
	Unknown	MFLU 16-1372	MF173608	MF173609	–	Tennakoon et al. 2021
Neopestalotiopsisprotearum*	Africa (Zimbabwe)	CBS 114178	JN712564	JN712498	KM199463	Maharachchikumbura et al. 2014
*N.rosae*	Oceania (New Zealand)	CBS 101057	KM116245	KM199359	KM199429	Maharachchikumbura et al. 2014
*Pestalotiopsisknightiae*	Oceania (New Zealand)	CBS 114138	KM116227	KM199310	KM199408	Maharachchikumbura et al. 2014
*P.malayana*	Southeast Asia (Malaysia)	CBS 102220	KM116238	KM199306	KM199411	Maharachchikumbura et al. 2014
*P.spathuliappendiculata*	Oceania (Australia)	CBS 144035	MH554366	MH554172	MH554845	Liu et al. 2019
*Pseudopestalotiopsiscocos*	Southeast Asia (Indonesia)	CBS 272.29	KM116276	KM199378	KM199467	Maharachchikumbura et al. 2014
Ps.theae*	East Asia (China), Southeast Asia (Thailand)	MFLUCC 12-0055	KM116282	JQ683727	JQ683711	Maharachchikumbura et al. 2014
*Robillardaafricana*	Africa (South Africa)	CBS 122.75	KR873281	KR873253	MH554656	Crous et al. 2015
*R.roystoneae*	East Asia (China)	CBS 115445	KR873282	KR873254	KR873317	Crous et al. 2015
	East Asia (China)	MFLUCC 19-0060	MW114402	MW114323	–	Tennakoon et al. 2021
R.sessilis*	Europe (Germany)	CBS 114312	KR873284	KR873256	KR873319	Crous et al. 2015
*R.terrae*	South Asia (India)	CBS 587.71	KJ710459	KJ710484	MH554734	Crous et al. 2015
*Seimatosporiumazaleae*	Unknown	MAFF 237478	AB593730	AB594798	AB594189	Tanaka et al. 2011
*S.biseptatum*	Oceania (Australia)	CPC 13584	JN871208	JN871199	MH554749	Barber et al. 2011
*S.botan*	America (Chile)	HMUC 316PD	–	JN088483	–	Díaz et al. 2012
*S.cornicola*	Europe (Italy)	MFLUCC 14-0448	–	KU974967	–	Wijayawardene et al. 2016
*S.cornii*	Europe (Italy)	MFLUCC 14-1208	KT868531	KT868532	–	Perera et al. 2016
*S.elegans*	Oceania (Australia)	NBRC 32674	AB593733	AB594801	MH554683	Tanaka et al. 2011
*S.eucalypti*	Africa (South Africa)	CPC 156	JN871209	JN871200	MH704627	Barber et al. 2011
*S.falcatum*	Oceania (Australia)	CPC 13578	JN871213	JN871204	MH554668	Barber et al. 2011
*S.grevilleae*	Africa (South Africa)	ICMP 10981	AF382372	AF405304	–	Jeewon et al. 2002
*S.italicum*	Europe (Italy)	MFULCC 14-1196	NG_064463	NR_157485	–	Hyde et al. 2017
*S.leptospermi*	Oceania (New Zealand)	ICMP 11845	AF382373	–	–	Jeewon et al. 2002
*S.obtusum*	Oceania (Australia)	CPC 12935	JN871215	JN871206	MH554669	Barber et al. 2011
*S.physocarpi*	Europe (Russia)	MFLUCC 14-0625	KT198723	KT198722	MH554676	Norphanphoun et al. 2015
*S.pistaciae*	West Asia (Iran)	CBS 138865	KP004491	KP004463	MH554674	Norphanphoun et al. 2015
*S.pseudorosae*	Europe (Italy)	MFLUCC 14-0468	KU359035	–	–	Li et al. 2015
*S.pseudorosarum*	Europe (Italy)	MFLUCC 14-0466	KT281912	KT284775	–	Ariyawansa et al. 2015
S.rosae*	Europe (Russia)	MFLUCC 14-0621	KT198727	KT198726	LT853253	Norphanphoun et al. 2015
*S.rosicola*	Europe (Italy)	MFLU 16-0239	MG829069	MG828958	–	Wanasinghe et al. 2018
	Europe (Italy)	MFLUCC 15-0564	MG829070	MG828959		Wanasinghe et al. 2018
*S.sorbi*	Europe (Italy)	MFLUCC 14-0469	KT281911	KT284774	–	Ariyawansa et al. 2015
*S.tostum*	Unknown	NBRC 32626	AB593727	AB594795	–	Rossman et al. 2016
*S.vaccinii*	Oceania (New Zealand)	ICMP 7003	AF382374	–	–	Jeewon et al. 2002
*S.vitis*	Europe (Italy)	MFLUCC 14-0051	KR920362	NR_156595	–	Senanayake et al. 2015
*S.walkeri*	Oceania (Australia)	CPC 17644	JN871216	JN871207	MH554769	Barber et al. 2011
*Seiridiumcancrinum*	Africa (Kenya)	CBS 226.55 = IMI 052256	MH554241	LT853089	LT853236	Liu et al. 2019
*Seir.cupressi*	Africa (Kenya)	CBS 224.55 = IMI 052254	MH554240	LT853083	LT853230	Liu et al. 2019
*Seir.eucalypti*	Oceania (Australia)	CBS 343.97	MH554251	MH554034	MH554710	Liu et al. 2019
*Seir.kartense*	Oceania (Australia)	CBS 142629 = CPC 20183	–	LT853100	LT853247	Liu et al. 2019
*Seir.kenyanium*	Africa (Kenya)	CBS 228.55 = IMI 052257	MH554242	LT853098	LT853245	Liu et al. 2019
Seir.marginatum*	Europe (Austria)	CBS 140404	–	KT949916	–	Jaklitsch et al. 2016
	Europe (France)	CBS 140403	MH554223	KT949914	LT853249	Liu et al. 2019
*Seir.neocupressi*	Europe (Italy)	CBS 142625 = CPC 23786	MH554329	LT853079	LT853226	Liu et al. 2019
*Seir.papillatum*	Oceania (Australia)	CBS 340.97	DQ414531	LT853102	LT853250	Liu et al. 2019
*Seir.phylicae*	Tristan da Cunha (Atlantic islands)	CBS 133587 = CPC 19964	–	LT853091	LT853238	Liu et al. 2019
*Seir.pseudocardinale*	Europe (Portugal)	CBS 122613 = CMW 1648	MH554206	LT853096	LT853243	Liu et al. 2019
*Seir.unicorne*	Oceania (New Zealand)	CBS 538.82 = NBRC 32684	MH554269	LT853088	LT853235	Liu et al. 2019
Strickeriakochii*	Europe (Austria)	C143	KT949918	KT949918	–	Jaklitsch et al. 2016
St.kochii*	Europe (Austria)	C149	KT949920	KT949920	–	Jaklitsch et al. 2016
*Phlogicylindriumuniforme*	Oceania (Australia)	CBS 131312	JQ044445	JQ044426	MH704634	Crous et al. 2015

Type species are marked with an asterisk ‘^*^’. New species are given in bold.

Phylogenetic analyses were performed using the CIPRES Science Gateway V.3.3 (https://www.phylo.org/). For maximum likelihood (ML) analyses, we used RAxML-HPC2 on XSEDE (8.2.12). *Phlogicylindriumuniforme* (CBS 131312) was selected as the outgroup taxon. One thousand non-parametric bootstrap iterations were performed using the “GTRGAMMA” algorithm. For Bayesian analysis, jModelTest2 on XSEDE (2.1.6) was used to estimate the best-fitting model for the combined LSU, ITS and *tub2* genes, and the GTR+I+G model was the best fit. In MrBayes on XSEDE (3.2.7a), four simultaneous Markov chains were run for 2,000,000 generations; trees were sampled and printed every 2,000 generations. The first 25% of all trees were submitted to the burn-in phase and discarded, while the remaining trees were used to compute posterior probabilities in the majority rule consensus tree (Cai et al. 2006, 2008; Wu et al. 2011; Zeng et al. 2019).

### ﻿Divergence time estimations

In this study, two secondary calibration nodes for the divergence time estimation of *Ciliochorella* were implemented to calibrate the tree: Node 1 was composed of *Phlogicylindrium* (outgroup, Phlogicylindriaceae) and 10 genera from the Sporocadaceae, which diverged 76 Mya; for Node 2 we used *Discosia*, *Robillarda* and the other seven genera (*Ciliochorella*, *Neopestalotiopsis*, *Pestalotiopsis*, *Pseudopestalotiopsis*, *Seimatosporium*, *Seiridium* and *Strickeria*), which diverged 44 Mya (Samarakoon et al. 2016). A maximum likelihood (ML) tree was used as input data and the data were analyzed via R8S 1.81 (https://sourceforge.net/projects/r8s/). The divergence time of *Ciliochorella* was estimated based on the PL (Penalized likelihood) method and TN algorithm (truncated Newton algorithm) obtained via R8S (Fig. 2). The R8S program only needs the second calibration node to estimate divergence times, and some methods in this program, such as NPRS (Nonparametric rate smoothing) and PL, which were first proposed by the author, are currently challenging to implement in similar software programs (Sanderson 2003). The PL method and TN algorithm have been implemented to estimate divergence times using data from the ML tree and secondary calibration nodes (Sanderson 2003). The ancient map is based on the results of Peng et al. (2023).

### ﻿Reconstruction of ancestral biogeographic

RASP (http://mnh.scu.edu.cn/soft/blog/RASP) was used to reconstruct the ancestral biogeography in this study. It is a tool to infer the ancestral state using S-DIVA (Statistical Dispersal-Vicariance Analysis), Lagrange (DEC), Bayes-Lagrange (S-DEC), BayArea, BBM (Bayesian Binary MCMC), Bayestraits and BioGeoBEARS packages (Yu et al. 2015, 2020). Members of *Ciliochorella* were coded based on their collection locality according to references (Table 1). Based on the distribution data in the table, six geographic regions were defined: A = Asia, B =America, C = Europe, D = Africa, E = Oceania, F = Tristan da Cunha, G = Unknown, using species from Asia, America, Europe, Africa, Oceania and Tristan da Cunha. In MrBayes on XSEDE (3.2.7a), chains were run for 2,000,000 generations; trees were sampled and printed every 2,000 generations. RASP 4.2 was used to reconstruct the ancestral state, and the most-optimal model was BAYAREALIKE.

### ﻿Screening of cellulase and laccase production

Cellulase screening was performed by the Congo red test (Liu and Fan 2012). A fungal cake with a diameter of 8 mm was isolated from the edge of the 7-day old colony and inoculated on solid PDA medium. After 7 days of inoculation, the culture was stained with 1 mg/mL Congo red solution for 10 min, and washed and fixed with 1 mol/L NaCl for 30 min.

Screening for laccase activity in the lignin peroxidase system requires the use of guaiacol-PDA solid medium (Wang et al. 2016), including PDA medium 40.20 g, agar 3.00 g, guaiacol 0.40 mL and distilled water to 1 L with 121 °C sterilization 30 min. The obtained strain was cultured at 26 °C for 7 days, and the fungal cake with a diameter of 8 mm was taken from the growing mycelium at the edge of the colony and inoculated on solid guaiacol-PDA medium. The growth of the strain was observed for 7 days of inoculation.

The supernatant was incubated on a shaker (150 rpm) for 12 hours at 26 °C, followed by centrifugation at 12,000 rpm to obtain a crude enzyme solution. The Thermo Varioskan Flash multifunctional enzyme reader has a characterized absorption peak at 540 nm, which can be used to assess cellulase activity based on changes in absorbance values. Laccase activity was characterized by the change in absorbance at 420 nm and the enzyme activity of the crude enzyme liquid was determined using the Laccase Activity Detection Kit (www.boxbio.cn). The experiment was repeated three times.

## ﻿Results

### ﻿Phylogenetic analyses

**Figure 1.**
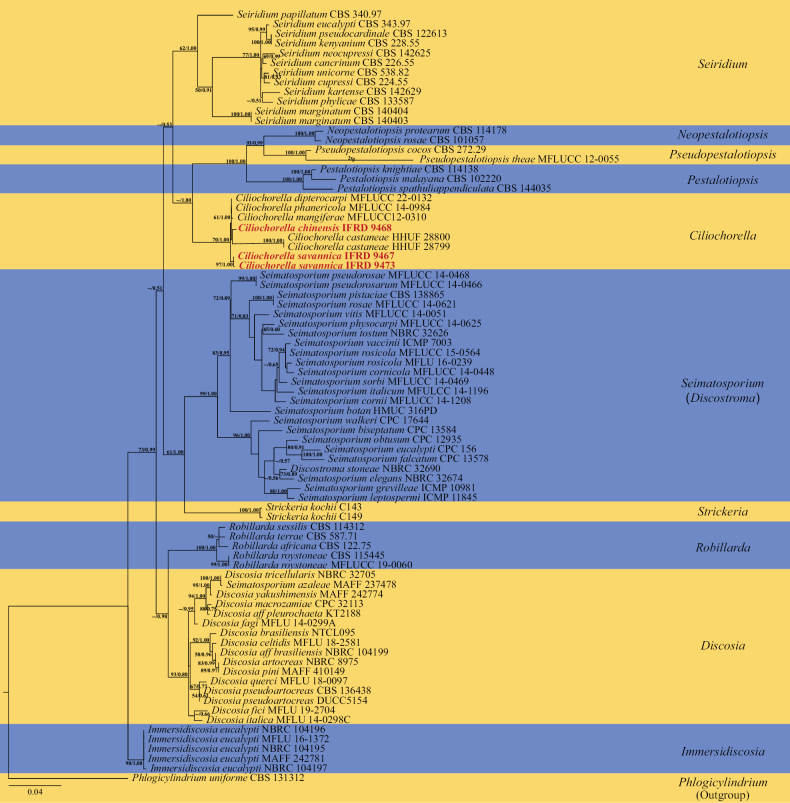
Phylogenetic tree of maximum likelihood analyses showing the relationships of *Ciliochorella* species based on combined LSU, ITS and *tub2* data set analysis. Bootstrap values of maximum likelihood values greater than 50% are shown on the left, while values for Bayesian posterior probabilities greater than 0.5 are shown on the right. *Discostroma* is the sexual morph of *Seimatosporium*. New species are shown in bold and red, followed by their strain number.

We analyzed a three-locus (LSU, ITS, *tub2*) data set of *Ciliochorella*. This data set consists of 203 sequences, including 75 LSU sequences, 75 ITS sequences and 53 *tub2* sequences from 80 taxa. The concatenated sequences have 2338 characters including gaps. The two topological trees obtained by maximum likelihood (ML) and Bayesian were found to be similar, and the best-scoring RAxML tree was used as the representative tree (Fig. 1). Bootstrap values of ML greater than 50% are shown on the phylogenetic tree, while values of Bayesian posterior probabilities greater than 0.5 are shown on the tree (Fig. 1).

Phylogenetic analysis showed that *Ciliochorella* species formed a clade with bootstrap values of 70% (in ML analysis) and Bayesian posterior probability of 1.00 (as a result of new species, the genus forms a separate clade). *Pestalotiopsis*, *Pseudopestalotiopsis*, and *Neopestalotiopsis* formed a clade with bootstrap values of 100% and Bayesian posterior probabilities of 1.00. Notably, this clade was adjacent to the *Ciliochorella* clade. In addition, *Ciliochorella* was also close to *Seiridium* (Fig. 1).

*Ciliochorellasavannica* is distinguished from other *Ciliochorella* in the phylogenetic tree and has a high support rate with 97% ML and 1.00 Bayesian posterior probabilities. *Ciliochorellachinensis* has a close relationship with *C.castaneae* (HHUF 28800).

### ﻿Divergence time estimation

**Figure 2.**
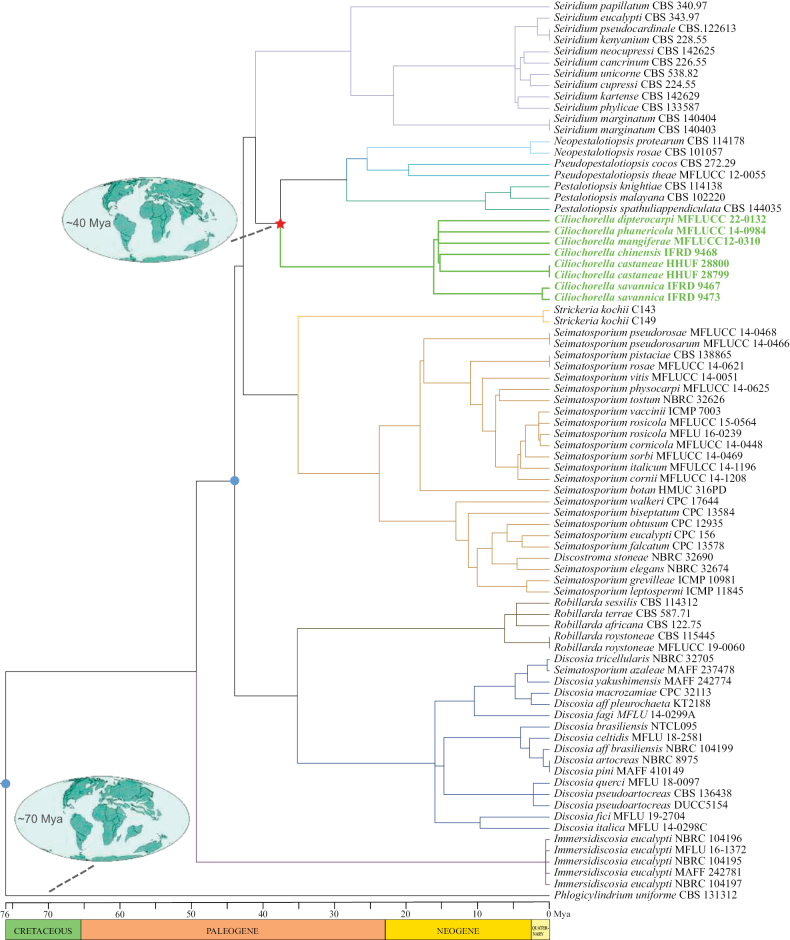
Divergence time tree based on ML analysis. Divergence times of all nodes were estimated by R8S software using two calibration points. The blue circles and the red star indicate secondary points and the divergence time of *Ciliochorella* respectively. *Ciliochorella* species are shown in bold and green. Maps were adopted from Peng et al (2023).

According to divergence time estimates (Fig. 2), the age of *Ciliochorella* is about 38 Mya in the Paleogene period and falling in the recommended divergence times of Xylariomycetidae by Samarakoon et al. (2016). The ten genera of Sporocadaceae were all originated in the Paleogene. The genus of *Immersidiscosia* initially diverged about 49 Mya. *Discosia* and *Robillarda* formed one clade, with a divergence of time about 35 Mya. The other seven genera formed one clade: *Neopestalotiopsis* diverged about at 25 Mya, divergence times of *Pestalotiopsis* in the analysis is about 28 Mya, *Pseudopestalotiopsis* diverged about at 25 Mya, *Seimatosporium* diverged about at 35 Mya, *Seiridium* diverged about at 41 Mya and *Strickeria* diverged about at 35 Mya.

### ﻿Ancestral biogeographic reconstruction analysis for *Ciliochorella*

**Figure 3.**
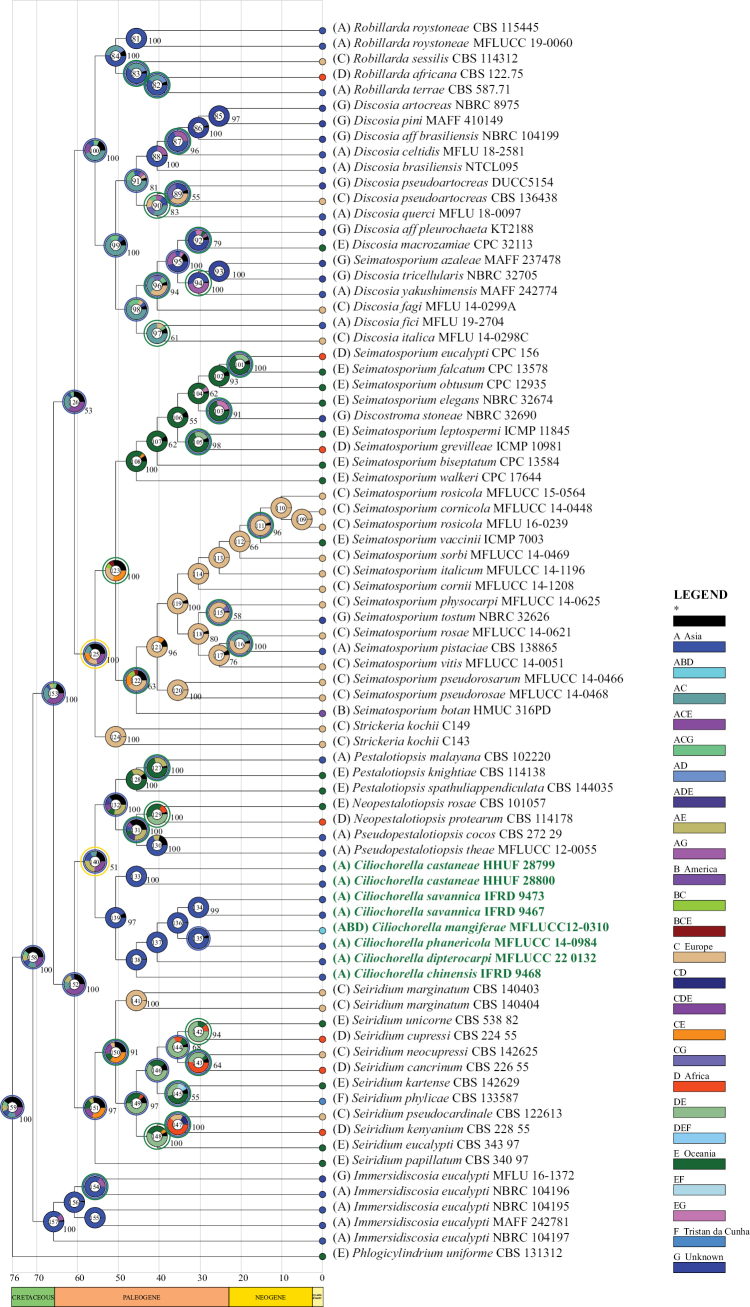
Ancestral biogeographic reconstructions are based on Bayesian trees. Each event is represented by a node number. Bayesian posterior probabilities are shown (≥ 50). A colored circle near the number at the nodes indicates the following: blue represents dispersal, green represents vicariance, and yellow represents extinction. *Ciliochorella* species are shown in bold and green.

Analysis of ancestral biogeographic reconstructions revealed that *Ciliochorella* species originated in Asia (Fig. 3, node 139). Dispersal, vicariance, extinction, and other historical events affected the biogeographic distribution of individual species. The evolutionary history of the ancestors of the genus *Ciliochorella* showed that the species of this genus underwent 45 dispersals, 27 vicariances, and 2 extinctions (Fig. 3, the blue circle represents dispersal, the green circle represents vicariance, and the yellow circle represents extinction). From approximately the Middle Paleogene, dispersal and vicariance events were frequent. In the early Paleogene, dispersal and extinction events occurred among ancestors of *Pestalotiopsis*, *Pseudopestalotiopsis*, *Neopestalotiopsis*, and *Ciliochorella*. After these events, *Ciliochorella* began to evolve independently of other genera (Fig. 3, node 140). *Pestalotiopsis*, *Pseudopestalotiopsis*, and *Neopestalotiopsis* are predicted to share the same ancestral biogeographic area (Fig. 3, node 132). Dispersal events occurred two times with *Ciliochorella* in the late Paleogene (about 30 Mya), which was followed by a period where *Ciliochorella* began spreading in Africa and America (Fig. 3, node 135). The result of the ancestral biogeographic reconstruction supported the notion that the Eurasian continent was the center of origin for *Ciliochorella*: the estimated ancestral distributions for nodes of the complex and its clades included both Asia and Europe (Fig. 3, node 139).

### ﻿Taxonomy

#### ﻿ Ciliochorella

Taxon classificationFungiAmphisphaerialesSporocadaceae

Syd., in Sydow & Mitter, Annls mycol. 33(1/2): 62 (1935)

B4E9FCF8-24C0-5993-9A4F-E9AF7C03F85C

Fungal Names: FN 7657

##### Type species.

*Ciliochorellamangiferae* Syd., Annls Mycol. 33(1/2): 63 (1935). Fungal Names: FN 270484.

##### Notes.

*Ciliochorella* is an asexually typified genus. Most species of this genus are saprophytic with the exception of *Ciliochorellacastaneae* Munjal. The conidiomata of *Ciliochorella* species are generally round, semi-immersed, and longitudinally lenticular. A prominent feature observed during the early stage of germination is apical and basal cells of conidia-produced germ tubes, and a vacuolated state of the protoplasm (Masilamani and Muthumary 1994). Conidiophores arise from the thin-walled, and are almost colorless cells of the basal or basal and parietal tissue, mostly reduced to conidiogenous cells. Occasionally, they are sparsely septate, branched or unbranched, colorless, smooth, invested in mucus (Sutton 1980; Nag Raj 1993). Conidiogenous cells are discrete, ampulliform, or conical with a long neck, colorless, and smooth. Conidia are cylindrical, straight, or slightly curved with septate pale brown middle cells and colorless end cells with appendages at one or both ends (Lee et al. 2006; Allegrucci et al. 2011; Tangthirasunun et al. 2015; Hyde et al. 2016; Wijayawardene et al. 2016; Liu et al. 2019a).

There are four *Ciliochorella* species for which molecular data is available on the NCBI repository (i.e. *C.castaneae*; *C.dipterocarpi* Samaradiwakara, Lumyong & K.D. Hyde; *C.mangiferae* and *C.phanericola* Norph., T.C. Wen & K.D. Hyde). *Ciliochorellamangiferae* is the earliest recorded species and described by Sydow and Mitter (1935) as the type species of this genus. Tangthirasunun et al. (2015) discovered a new record of *C.mangiferae* in Thailand and provided some its molecular data for this species. The first discovery of *C.castaneae* was in India (Nag Raj 1993), but Endo et al. (2008) added a new record for this species in Japan and also added molecular data. Samaradiwakara et al. (2023) discovered *C.dipterocarpi* and analyzed the species molecularly. For the other species of this genus, there is still no molecular data available, and comprise *C.splendida* Nag Raj & R.F. Castañeda and *C.buxifoliae* Allegr., Ellegr. & Aramb (Nag Raj 1993; Allegrucci et al. 2011). The morphological characteristics of all *Ciliochorella* species are provided in Table 2.

**Table 2.** The comparison of micro-morphological characteristics of *Ciliochorella*.

Species	Host-Substratum	Conidiomata diam (μm)	Conidiomata with a papillary	Conidia (μm)	Mean conidium length/width ratio	Basal appendages number	Reference
*Ciliochorellabuxifoliae*	*Scutiabuxifolia*	300–500	–	19–21×2.5–2.7	7:1	1	Allegrucci et al. 2011
*C.castaneae*	*Castaneaeuropaea*	450–650	Yes	13–19×2.5–3.2 (Ave.16.0×3.0)	11.1:1	1	Nag Raj 1993; Endo et al. 2008
*C.chinensis*	Unidentified leaf litter	894–1314	Yes	13.9–17.9×3.3–4.1 (Ave.15.7×3.6)	4.4:1	1	In this study
*C.dipterocarpi*	*Dipterocarpaceaealatus*	650–800	No	9–18×1–3 (Ave.14×2)	7:1	1	Nethmini et al. 2023
*C.mangiferae*	*Mangiferaindica*	400–800	–	32–43×2.5–3.5 (Ave. 37×3)	12.3:1	1	Nag Raj 1993
*C.phanericola*	*Phanerapurpurea*	1000–1200	No	13–15×2.8–3.5 (Ave. 15×3.7)	4.1:1	1	Hyde et al. 2016
*C.savannica*	Unidentified leaf litter	530–952	Yes	11–16×2–3 (Ave.14×2.6)	5.4:1	0	In this study
*C.splendida*	*Quercusoleoidessubsp.Sagrana*	–	–	24–40×2.5–3 (Ave. 32×2.7)	11.8:1	1	Nag Raj 1993

#### ﻿ Ciliochorella chinensis

Taxon classificationFungiAmphisphaerialesSporocadaceae

H.X. Wu & J.C. Li sp. nov.

0E4DA6CA-01DA-5960-A8D9-7C916C24A152

Fungal Names: FN 571291

##### Etymology.

The species epithet reflects China where the species of *Ciliochorella* was first collected country.

##### Holotype.

IFRD9468.

##### Description.

**Figure 4.**
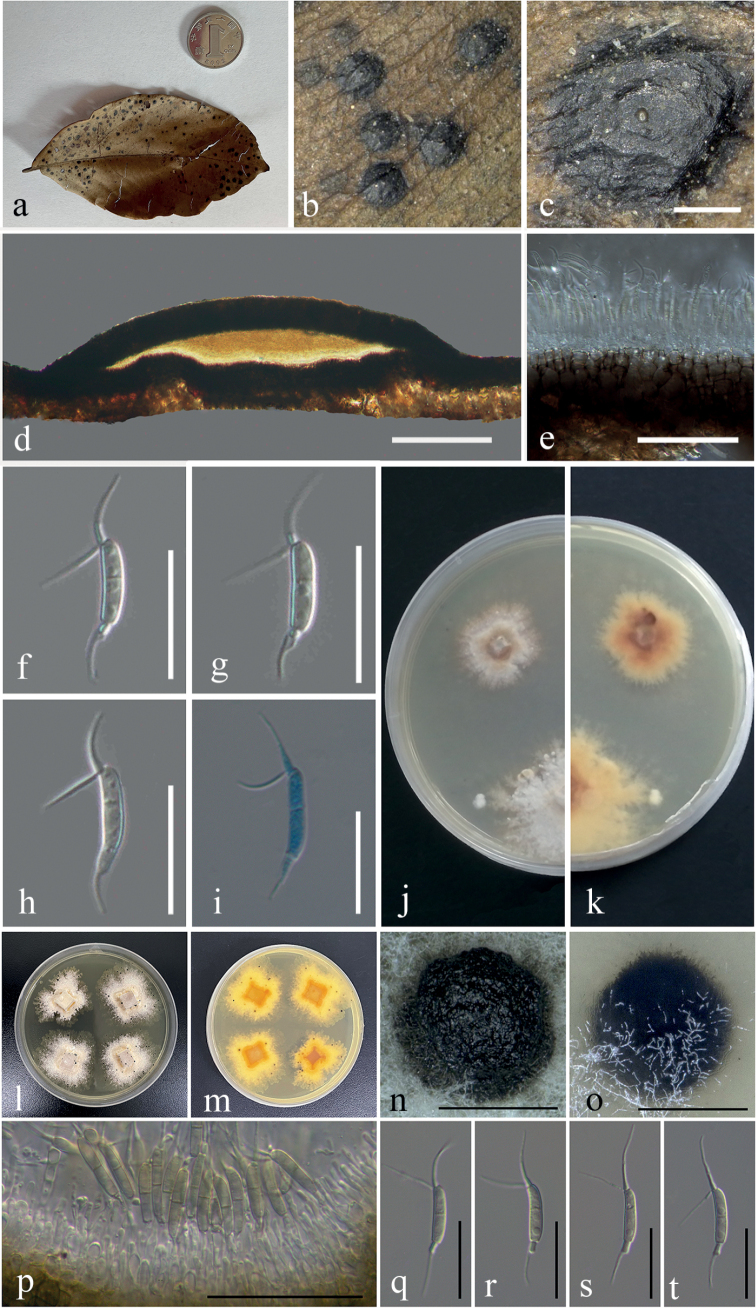
*Ciliochorellachinensis* (IFRD9468, holotype; IFRDCC3202, ex-type strain) **a, b** the specimen **c** surface of fruiting bodies **d** longitudinal section of the conidioma **e** peridium **f–h** mature conidia **i** mature conidia in cotton blue **j, k** colonies on PDA (k from below) **l, m** colonies on PDA (m from below) **n** fruiting bodies on PDA**o** fruiting bodies in PDA**p** peridium **q–t** mature conidia. Scale bars: 400 µm (**c, n, o**); 200 µm (**d**); 40 µm (**p**); 20µm (**e**); 10 µm (**f–i, q–t**).

Saprobic on leaf litter. ***Asexual morph***: Coelomycetous. ***Conidiomata*** 894–1314 μm diameter (x¯ = 1055 μm, n = 14), unilocular, semi-immersed, circular areas, dark brown, mostly aggregated, sometimes solitary, forming a papilla in the center (Fig. 4a–c). ***Conidiomata wall*** comprises a few to several layers of cells of *textura angularis*, with the innermost layer thin, transparent, and precisely arranged, the outer layer dark brown to black (Fig. 4d). ***Conidiophores*** appear to be reduced to conidiogenous cells. ***Conidiogenous cells*** are enteroblastic phialidic, formed from the innermost layer of the wall, hyaline to pale brown, and smooth (Fig. 4e). ***Conidia*** 14–18 × 3–4 μm (x¯ = 15.7 × 3.6 μm, n = 12), excluding apical and basal appendages, mean conidium length/width ratio = 4.4:1, navicular to subcylindrical, slightly curved, 1-septate, wide middle two cells with apical cell transformed into two forked filiform cellular appendages, 9–16 μm (x¯ = 12.5 μm, n = 20), the narrow basal cell with basal appendage, 4–7 μm (x¯ = 5.4 μm, n = 11), colorless to light brown, with guttules on the conidia surface (Fig. 4f–i). ***Sexual morph***: Unknown.

##### Culture characteristics.

Colonies on PDA, reaching 4.4 cm (n = 3) diam after 7 days at 26 °C, producing dense mycelium, irregular circular, margin rough, white (Fig. 4j, k). Conidia germinated and grew deep into the medium. There was a clear boundary between the center and the most marginal part. The culture grew fruiting bodies after about four months on PDA medium at 26 °C (Fig. 4l, m). The morphology of conidiophores and conidia in the semi-immersed or fully embedded medium was consistent with that found under natural conditions (Fig. 4n–t).

##### Material examined.

China. Yunnan Province, Yuanjiang County, Yuanjiang National Nature Reserve (Xiaohedi), on dead leaves of an unidentified plant, 23°28'33"N, 102°21'1"E, elevation 423 m, June 2021, Hai-Xia Wu, Jin-Chen Li, and Xin-Hao Li (IFRD9468, **holotype**; IFRDCC3202, **ex-type**).

##### Notes.

The phylogenetic tree shows that *Ciliochorellachinensis* has a close relationship with *C.castaneae* (HHUF 28800) (Fig. 1). A BLAST search conducted within GenBank, the match for LSU showed a 98.72% similarity to *C.castaneae* (HHUF 28800, this species only has LSU) across a query coverage of 95%. At present, the phylogenetic relationship in this genus is not comprehensive enough, so the classification depends greatly on their morphology. Morphologically, the conidiomata of *C.chinensis* have a papillary, which the conidiomata of *C.phanericola* lack. The conidiomata of both species display different sizes (Table 2).

#### ﻿ Ciliochorella savannica

Taxon classificationFungiAmphisphaerialesSporocadaceae

H.X. Wu & J.Y. Song sp. nov.

D2961BE1-60EB-5B3D-B83B-900F8C12B8F4

Fungal Names: FN 571290

##### Etymology.

Epithet derived from the type locality (Yuanjiang Savanna Ecosystem Research Station).

##### Holotype.

IFRD9467.

##### Description.

**Figure 5.**
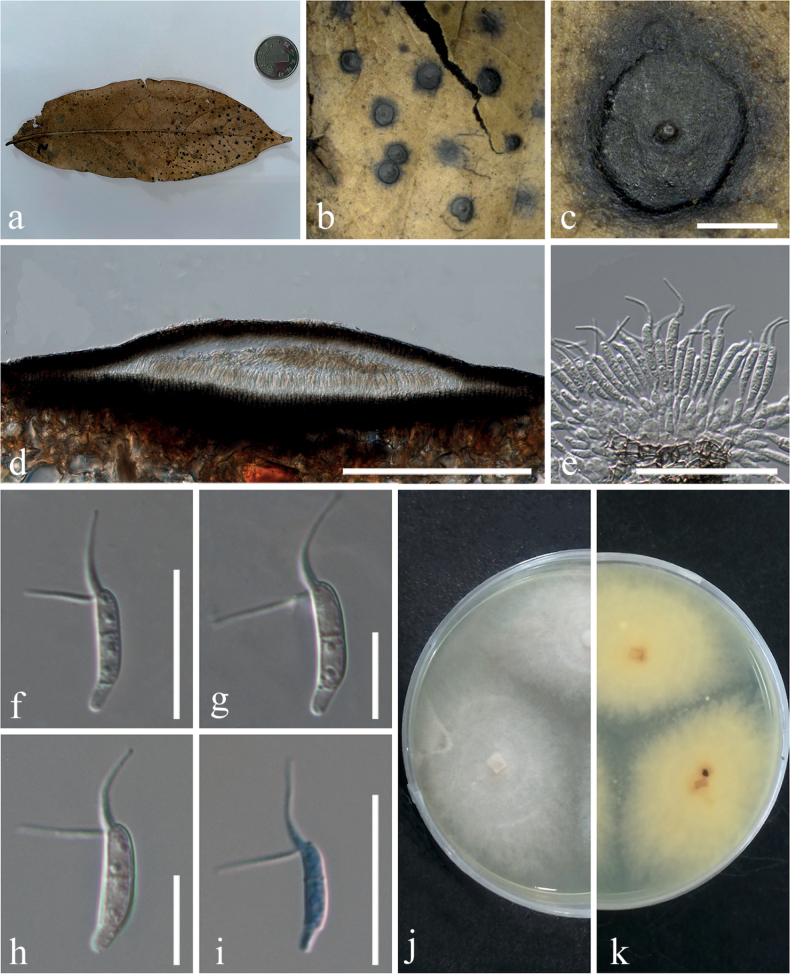
*Ciliochorellasavannica* (IFRD:9467, holotype; IFRDCC:3201, ex-type) **a, b** the specimen **c** surface of fruiting bodies **d** longitudinal section of the conidioma **e** peridium **f–h** mature conidia **i** mature conidia in cotton blue **j, k** colonies on PDA (k from below). Scale bars: 400 µm (**c**); 200 µm (**d**); 20µm (**e**); 10 µm (**f–i**).

***Saprobic*** on leaf litter. ***Asexual morph***: Coelomycetous. ***Conidiomata*** 530–950 μm diameter (x¯ = 758 μm, n = 23), unilocular, semi-immersed, circular areas, black, mostly aggregated, sometimes solitary, with a papilla central circular ostiole (Fig. 5a–c). ***Conidiomata wall*** comprises a few to several layers of cells of *textura angularis*, with the inner layer being mostly thin, brown, whereas the outer layer appears dark brown to black. The longitudinal section is lenticular, the base is well developed (Fig. 5d). ***Conidiophores*** are reduced to conidiogenous cells. ***Conidiogenous cells*** enteroblastic phialidic, formed from the innermost layer of the wall, hyaline to pale brown, smooth (Fig. 5e). ***Conidia*** 11–16 × 2–3 μm (x¯ = 14 × 2.6 μm, n = 22) excluding apical appendages, mean conidium length/width ratio = 5.4:1, navicular to subcylindrical, slightly curved, 1-septate, narrow basal cell, wide middle two cells with apical cell transformed into two forked filiform cellular appendages 7–13 μm (x¯ = 10 μm, n = 22), 2–4-guttulates on the surface of the conidia, without basal appendages (Fig. 5f–i). ***Sexual morph***: Unknown.

##### Culture characteristics.

Conidia germinated and hyphae grew in emission form the center to the outside (Fig. 5j, k). Colonies growing on PDA, reaching a diameter of 4.4 cm (n = 3) after 7 days at 26 °C, producing dense mycelium, circular, margin rough. Surface white from the surrounding of the mycelium on PDA and pale yellow in reverse.

##### Material examined.

China. Yunnan Province, Yuanjiang County, Yuanjiang Savanna Ecosystem Research Station (Xishuangbanna Tropical Botanical Garden, Chinese Academy of Sciences), 23°28'31"N, 102°10'38"E, 579 m, on dead leaves of an unidentified plant, June 2021, Hai-Xia Wu, Jin-Chen Li, and Xin-Hao Li (IFRD9467, **holotype**; IFRD9473, **paratype**; IFRDCC3201, **ex-type)**.

##### Notes.

Two strains of *Ciliochorellasavannica* (holotype and paratype) correspond to *Ciliochorella* described by Sydow and Mitter (1935). The phylogenetic analysis showed that this species is only distantly related to other species in *Ciliochorella*. The number of different bases in ITS and LSU sequences of holotype and paratype was 7 (1033/1040) and 4 (1406/1410), respectively. These two strains of *C.savannica* formed a subclade within *Ciliochorella*, with 97% ML and 1.00 Bayesian posterior probabilities (Fig. 1). They differ morphologically from other species in conidiomata size (530–952 μm) and mean conidium length/width ratio (5.4:1) (Table 2). The significant characteristic is that *C.savannica* has conidia that lack basal appendages, whereas *Ciliochorella* species have this characteristic.

### ﻿Enzyme activity screening

**Figure 6.**
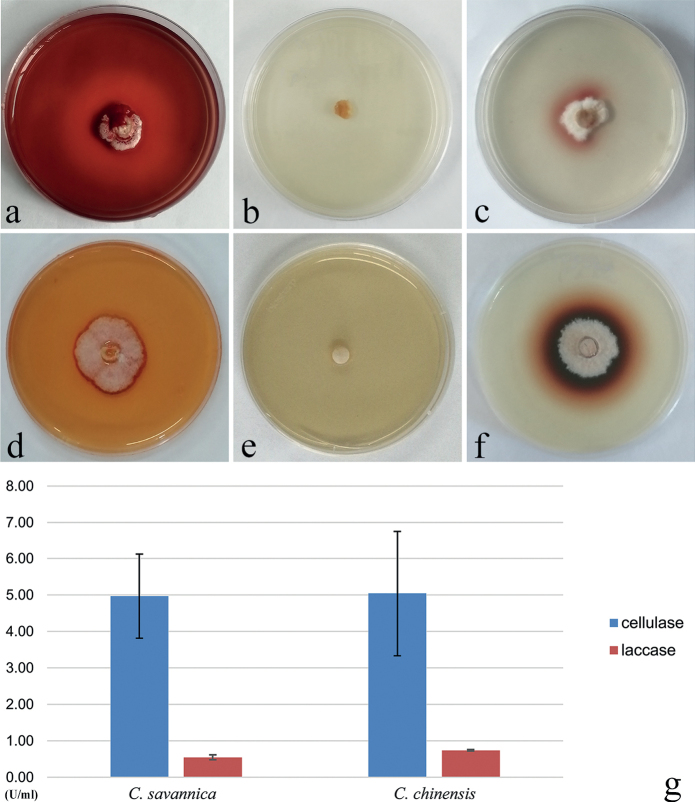
Screening of enzyme activity in culture **a***C.chinensis* was cultured on solid PDA medium for 7 days and stained with Congo red stain **b***C.chinensis* on solid guaiacol-PDA medium after 1 day **c***C.chinensis* on solid guaiacol-PDA medium after 12 days **d***C.savannica* was cultured on PDA solid medium for 7 days and stained with Congo red stain **e***C.savannica* on guaiacol-PDA solid medium 1 day **f***C.savannica* on solid guaiacol-PDA medium for 10 days **g** determination of enzyme activity.

The temperature was maintained at 26 °C. After 7 days of inoculation, Congo red staining was used to determine whether the strain had cellulase production ability (Fig. 6a, d). The results showed that both *C.chinensis* and *C.savannica* produced discoloring circles on the solid medium, with the discoloration of *C.savannica* being more pronounced. This indicates that cellulase plays a role in the cellulose degradation of both species.

Guaiacol-PDA solid medium was used to screen for laccase, and the temperature was set at 26 °C. The results were as follows: *C.chinensis* had a lighter color reaction on the solid medium until after 12 days (Fig. 6b, c) while *C.savannica* showed a color reaction obvious on the solid medium after 10 days (Fig. 6e, f).

The enzyme activity of the supernatant was determined by the Thermo Varioskan Flash enzyme marker. The average content of cellulose and laccase for *C.savannica* was 4.97 U/ml (n = 3) and 1.16 U/ml (n = 3); and for *C.chinensis*, the average cellulose content was 5.05 U/ml (n = 3) and laccase content was 1.71 U/ml (n = 3) (Fig. 6g). This suggests that laccase participates in lignin degradation of both species. There is no strong positive correlation between color reaction and numerical value, and the specific reasons need to be further explored.

## ﻿Discussion

*Ciliochorella* species play important roles in the decomposition of litter (Saparrat et al. 2010), and as such are a part of the carbon cycle throughout the world. The main body of *Ciliochorella* research has focused on the phylogeny and morphology of these fungi (Kalani 1963; Seshadri et al. 1969; Nag Raj 1993; Endo et al. 2008; Allegrucci et al. 2011; Hyde et al. 2016; Samaradiwakara et al. 2023). By contrast, the evolutionary history of *Ciliochorella*, whether based on DNA sequence analysis or the ecosystem studies, has received less attention and has remained unclear.

Wijayawardene et al. (2022b) stated that the subtropical to tropical regions in Asia will be an important region for discovering new fungal taxa, specifically asexually typified taxa. In China, the savanna-like vegetation has a unique geography and complex topography, which has contributed to the formation of various habitats with high biodiversity (Zhu and Tan 2022). Investigations have been carried out in the savanna-like vegetation in hot dry valleys of southwestern China, and this site is referred to as the “Chinesesavanna”. However, only a few reports have focused on fungi in this habitat, most of which have concentrated on endophytic and soil fungi (He et al. 2011; Ruan 2011; Yi et al. 2011). In addition, most *Ciliochorella* species were recorded from Asia except for *C.buxifoliae* and *C.splendida*, both of which occur in America (Nag Raj 1993; Endo et al. 2008; Allegrucci et al. 2011; Tangthirasunun et al. 2015; Hyde et al. 2016). According to the best of our knowledge, *Ciliochorella* species have not been recorded from China before. Our studies resulted in the discovery of two new *Ciliochorella* species: *C.chinensis* and *C.savannica*. This finding not only contributed two new species to the growing catalog of microfungal species found in the *Chinesesavanna*, but also represents the first *Ciliochorella* specimens reported for China. The two new species in this paper have been characterized based on phylogenetic analysis (Fig. 1) and morphological characteristics (Table 2). They belong to the genus *Ciliochorella*, which is part of the Sporocadaceae family, and the result is consistent with previous studies (Samarakoon et al. 2016; Liu et al. 2019a; Tennakoon et al. 2021).

The study of fossil fungi has become an essential tool for understanding fungal evolution and diversification, as well as elucidating the relationships of fungi to other organisms in the historical context of a given ecosystem (Taylor et al. 2015; Liu et al. 2019b; Samarakoon et al. 2019; Li et al. 2023). Despite wide distribution and large population, the majority of fungi (mycelia) are readily decomposed after death, resulting in a scarcity of fungal fossils that can be utilized for research concerning evolution (Tao and Chen 2020). In this report, we used the r8s program based on molecular clocks and two secondary calibration nodes to assess divergence times, which allowed us to estimate the emergence of the *Ciliochorella* at around 38 Mya during the Paleogene (Fig. 2). The Cretaceous-Paleogene mass extinction caused the disappearance of numerous groups, and its aftermath saw the rapid diversification of surviving species (Klein et al. 2021). According to the results of this study, *Ciliochorella* appeared in the middle and late periods of the Paleogene explosion of species. In addition, recent phylogeny and genomic studies used for the divergence time of Sordariomycetes indicate that relying solely on genus-level estimations may lack sufficient evidence and could potentially introduce errors (Chen et al. 2023). However, the divergence time estimated in this study does not conflict with the results based on genomic data.

The ancestral biogeography of *Ciliochorella* was investigated for the first time in this study. The result showed that the ancestor of *Ciliochorella* species originated from the Eurasian continent during the late Cretaceous. From approximately the late Cretaceous to the early Paleogene, there were some dispersal, vicariance and extinction events, which may be related to extreme climate incidents (Hu and Liu 2003). At about 30 Mya, there are two dispersal events that occurred within *Ciliochorella*. Up to this point, *Ciliochorella* species have been only found in Asia, Africa and America (Nag Raj 1993; Masilamani and Muthumary 1994; Endo et al. 2008; Tangthirasunun et al. 2015; Hyde et al. 2016). The results of our study clarify the evolutionary history of *Ciliochorella* ancestors and also provide a reference for the estimation of the divergence times of similar genera.

Some pathogenic plant fungi eliminate the effects of plant antiviral and tannic acid via laccase activity (Dong and Changsun 2021). By screening *Ciliochorellachinensis* and *C.savannica* strains on the medium, we found that both new species produce laccase and cellulase. They are involved in the decomposition of lignin and cellulose of leaf litter in their natural habitat, but their decomposition efficiency needs further study. Hyde et al. (2016) identify *C.phanericola* as a pathogen. The present study also supports the idea that *Ciliochorella* may have a potential role as a pathogenic plant fungus.

## Supplementary Material

XML Treatment for Ciliochorella

XML Treatment for Ciliochorella chinensis

XML Treatment for Ciliochorella savannica

## ﻿Additional information

### Author ORCIDs

Jia-Yu Song https://orcid.org/0000-0002-0884-7594

Hai-Xia Wu https://orcid.org/0000-0002-7169-6717

Jin-Chen Li https://orcid.org/0000-0001-8977-1829

Wei-Feng Ding https://orcid.org/0000-0002-2471-8071

Cui-Ling Gong https://orcid.org/0009-0005-9282-0974

Xiang-Yu Zeng https://orcid.org/0000-0003-1341-1004

Nalin N. Wijayawardene https://orcid.org/0000-0003-0522-5498

Da-Xin Yang https://orcid.org/0009-0008-9985-4669

### Data availability

All of the data that support the findings of this study are available in the main text.
